# Melatonin and the cardiovascular system in animals: systematic review and meta-analysis

**DOI:** 10.6061/clinics/2021/e2863

**Published:** 2021-09-28

**Authors:** Eduardo Carvalho de Arruda Veiga, Ricardo dos Santos Simões, Leonardo L. Caviola, Luiz Carlos Abreu, Ricardo Carvalho Cavalli, José Cipolla-Neto, Edmund Chada Baracat, José Maria Soares

**Affiliations:** I Departamento de Obstetricia e Ginecologia, Hospital das Clinicas HCFMRP-USP, Faculdade de Medicina de Ribeirao Preto, Universidade de Sao Paulo, Ribeirao Preto, SP, BR.; II Departamento de Obstetricia e Ginecologia, Hospital das Clinicas HCFMUSP, Faculdade de Medicina, Universidade de Sao Paulo, Sao Paulo, SP, BR.; III Disciplina de escrita cientifica, Faculdade de Medicina do ABC, Santo Andre, SP, BR.; IV Departamento de Fisiologia e Biofisica, Instituto de Ciencias Biomedicas (ICB-USP), Universidade de Sao Paulo, Sao Paulo, SP, BR.

**Keywords:** Cardiology, Melatonin, Meta Analysis, Review, Systematic Review

## Abstract

Melatonin, a hormone released by the pineal gland, demonstrates several effects on the cardiovascular system. Herein, we performed a systematic review and meta-analysis to verify the effects of melatonin in an experimental model of myocardial infarction. We performed a systematic review according to PRISMA recommendations and reviewed MEDLINE, Embase, and Cochrane databases. Only articles in English were considered. A systematic review of the literature published between November 2008 and June 2019 was performed. The meta-analysis was conducted using the RevMan 5.3 program provided by the Cochrane Collaboration. In total, 858 articles were identified, of which 13 were included in this review. The main results of this study revealed that melatonin benefits the cardiovascular system by reducing infarct size, improving cardiac function according to echocardiographic and hemodynamic analyses, affords antioxidant effects, improves the rate of apoptosis, decreases lactate dehydrogenase activity, enhances biometric analyses, and improves protein levels, as analyzed by western blotting and quantitative PCR. In the meta-analysis, we observed a statistically significant decrease in infarct size (mean difference [MD], -20.37 [-23.56, -17.18]), no statistical difference in systolic pressure (MD, -1.75 [-5.47, 1.97]), a statistically significant decrease in lactate dehydrogenase in animals in the melatonin group (MD, -4.61 [-6.83, -2.40]), and a statistically significant improvement in the cardiac ejection fraction (MD, -8.12 [-9.56, -6.69]). On analyzing potential bias, we observed that most studies presented a low risk of bias; two parameters were not included in the analysis, and one parameter had a high risk of bias. Melatonin exerts several effects on the cardiovascular system and could be a useful therapeutic target to combat various cardiovascular diseases.

## BACKGROUND

Melatonin (N-acetyl-5-methoxytryptamine) is a hormone produced by the pineal gland exclusively at night and is released into the bloodstream and cerebrospinal fluid in a circadian manner to regulate several physiological and neuroendocrine functions (1-3). The effects of melatonin are dependent on non-receptor- and receptor-mediated mechanisms of action. Membrane melatonin receptors (MT1, MTNR1A, MT1, and MTRN1B) are G-protein-coupled receptors, signaling through G_i_-G_0_ or G_q_-G_11_ transduction pathways, depending on the target organ. Melatonin secreted at night might interact with its effector and produce immediate effects when melatonin is present in the circulation (e.g., nighttime blood pressure dipping). Moreover, during the night and through several mechanisms of action, melatonin primes prospective effects (such as controlling autonomic nervous system activity) that can be observed only during the day when no pineal melatonin production occurs (3-6).

Over the last 20 years, several studies have suggested that melatonin influences the cardiovascular system (7,8). Melatonin may have significant anti-inflammatory and cardioprotective properties by directly eliminating free radicals, as well as indirectly via antioxidant activity. In addition, melatonin may be involved in blood pressure regulation and have significant anti-atherogenic effects (8-13).

In this systematic review, cardiovascular diseases such as hypertension, myocardial infarction, ischemia, and reperfusion were selected to verify the action of melatonin, as we believe that these cardiopathies currently represent a large number of cardiovascular diseases (13,14). Our study aimed to verify the effects of melatonin in an experimental model of myocardial infarction.

## SEARCH STRATEGIES

**Figure 1:**
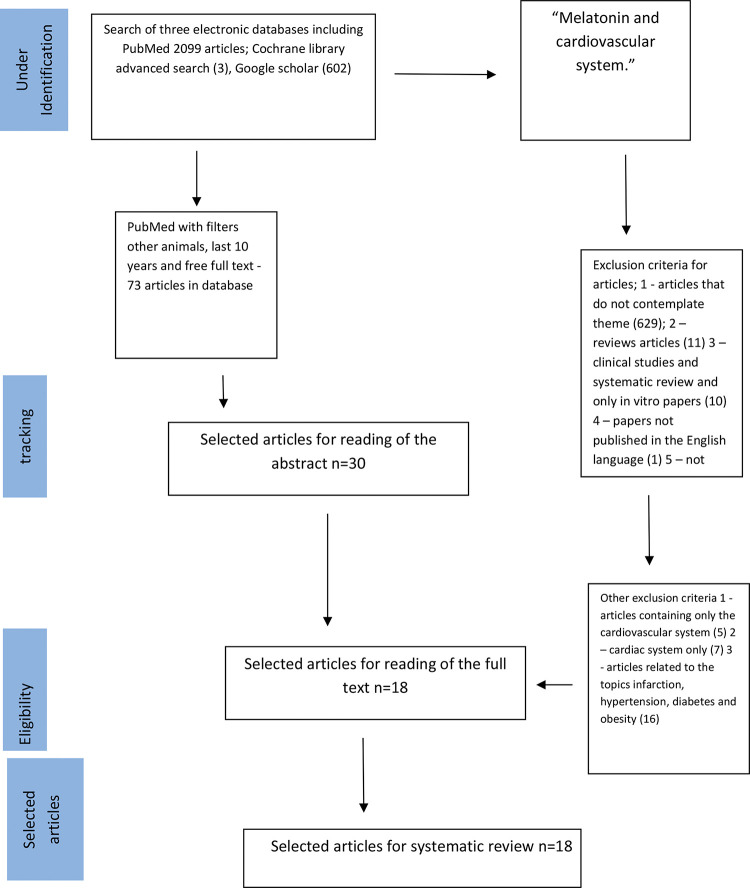
Flow chart of experimental design.

In the present study, the search strategy was performed as described by Tawfik et al. (15). We used MEDLINE, Google Scholar, and Cochrane databases and reviewed literature published from November 2008 to June 2019; we restricted this systematic review to the last ten years, covering the latest and most relevant articles worldwide. First, we selected keywords from related articles, using Medical Subject Headings (MeSH) to identify more related keywords with similar meanings as follows: (“melatonin”) [MeSH Terms] AND (“cardiovascular system”) [MeSH Terms] [All Fields]. We then searched the three databases. Accordingly, we identified 2096 articles in PubMed using the “other animals” filter, 602 articles using Google Scholar filtering for keywords only in the title, and three articles using a Cochrane Library advanced search; the terms used were “melatonin and cardiovascular system” In addition, we reviewed retrieved articles to identify additional studies (Figure 1). This review was conducted according to the recommendations of the Preferred Reporting Items for Systematic Reviews and Meta-Analysis (PRISMA) (16,17).

We excluded studies with cell culture experiments, as well as pre- and post-conditioning studies. The inclusion criteria were animal studies, cell culture studies, and *in vivo* experiments. The control group was the melatonin group in this study. The melatonin group varied in each article, as studies persistently experimented with a melatonin group related to a drug or an event.

The process of paper retrieval and titles and abstract evaluation was conducted by two independent blinded researchers capable of compiling systematic reviews (ECV and RS), following the inclusion and exclusion criteria according to the tenets of PICO (16-19). The PICO was defined as patients in case the systematic review was performed in animals, interventions considering the administration of melatonin in animals using an experimental model of myocardial infarction, comparison, to compare the melatonin group with the control group receiving no melatonin, and outcome, which were results of administering melatonin. The selected articles were critically evaluated to determine their potential inclusion in the review. In the event of a disagreement between investigators regarding studies selected, a third reviewer was consulted (LCA).

In the present systematic review, data obtained from selected studies were tabulated, and the following characteristics were listed when present in the articles: authors’ names, year of publication, animal type, sex (M/F), animal species, age (months), weight, induction model, and site injury (Table 1). Table 2 presents the following information: authors, sample size, number of groups, number of animals per group, melatonin administration, melatonin doses, and dependent variables. Table 3 lists the most frequent recommendations in preclinical research guidelines for *in vivo* animal experiments (18). Table 4 evaluates the study characteristics of selected controlled animal studies, with prior exercise and myocardial infarction as variables that showed a significant difference between the melatonin control group and the study group. These were classified as S for “significant difference,” and variables that did not present a significant difference were classified as NS (not significant).

RevMan (version 5.3; Cochrane Collaboration, Oxford, UK) was used to perform the meta-analysis. The random-effects model was used to account for the heterogeneity.

### Statistical analysis

Mean values and standard deviation between studies, presented as the mean difference (MD) of post-intervention values after calculating the inverse variance, were employed to verify the magnitude of the protection afforded by melatonin (19). In addition, heterogeneity was assessed using Cochran’s Q and I^2^ tests, followed by visual inspection of the graph. The analyses were performed using the RevMan software (version 3.3.1) (20).

## RESULTS

Figure 1 presents the search process, identification, and selection of articles. Based on our search strategies, 73 articles were retrieved from 2099 identified articles in PubMed using the “other animals” filter; among these, 18 were selected after reading the title and abstract. In addition, we selected three articles from BIREME and 0 articles from the Cochrane database. The inclusion and exclusion criteria are described in Figure 1 (21-38). Articles were primarily excluded when assessments were performed in human subjects, apart from being unrelated to components of PICO; we mainly focused on experimental animal studies.

In Table 4, we employed the criteria of Henderson et al. (18). We found that 61.11% (21,22,25-27,30,31,33,35,36,38) of selected studies had an appropriate sample size and 61.11% (21,23,25,26,27,30,31,33-36) had randomized animals, according to their materials and methods. All articles were blinded to the outcome assessment (21-38). We could not determine the criterion underlying the flow of animals through experiments, as no explicit statement regarding the same was available in the materials and methods. We observed that 77.77% of articles selected appropriate control groups (21,23,25-31,34,36) and 61.11% (23,25,26,28,30-32,34,36,38) had well-defined dose-response relationships. Moreover, all studies analyzed (21-38) had standard characterizations of animal properties at baseline. Overall, 89% of studies had employed an appropriate animal model that simulated the human manifestation of the disease (21-23,25-27,29-38). All studies that examined treatment responses according to a known mechanism (21-38) had characteristics within the requested standards. Only 67% (23-25,31,33,35,37,38) of selected manuscripts were within the range of the standard model of patient age in clinical settings. All studies did not follow other standard application models. In replicating different models of the same disease, 78% of studies (21-23,25-29,30,32,34-38) were independently replicated, whereas 88.88% (21-23,25-29,31-38) were replicated in different species. For studies where the objective was inter-study standardization of an experimental design, 39% (25-28,32,35,37) reached this standard (Table 4).

**Figure 2:**
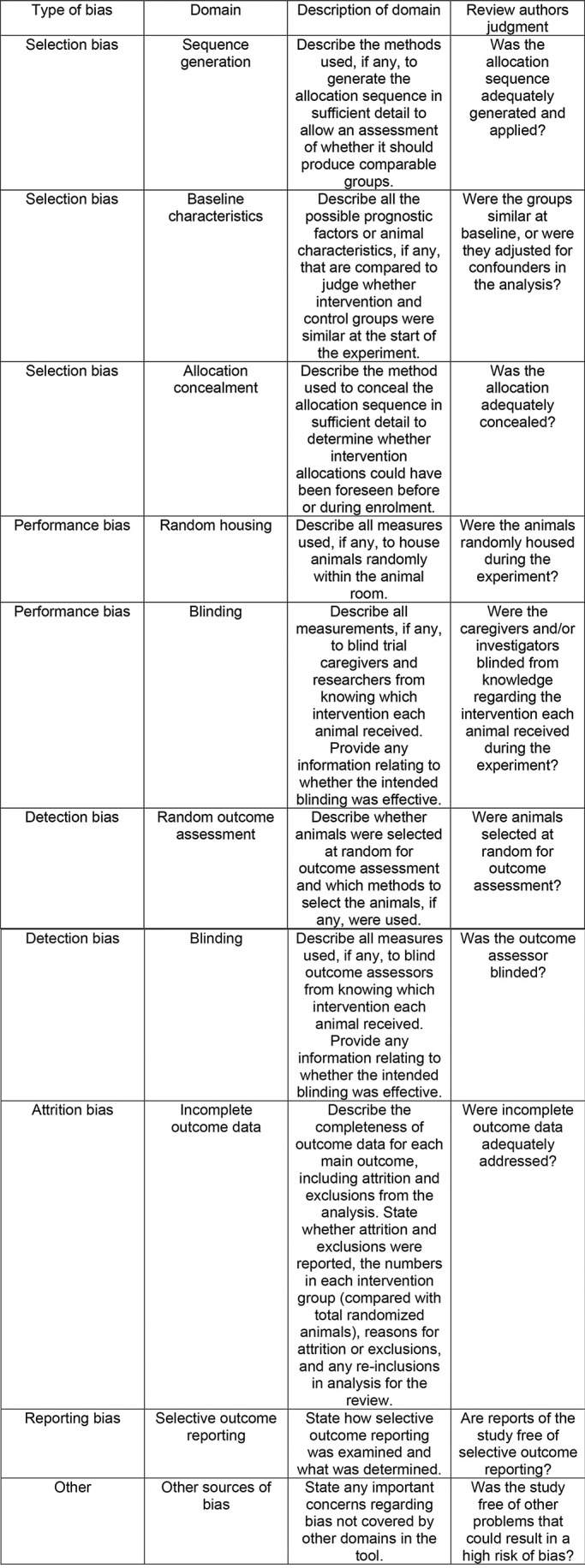
Representation of the SYRCLE’s risk of bias tool for animal studies. Hooijmans et al. (43).

In Table 5, we analyzed the study characteristics of selected controlled animal studies. Accordingly, we obtained the following results according to each experiment performed in articles examined in this systematic review. Table 5 presents experiments in which melatonin significantly improved the investigated variable (marked as S), as well as those where melatonin showed no significant improvements (NS). Chen et al. (23), Liu et al. (31), and Petrosillo et al. (38) reported that melatonin significantly decreased infarct size. Zhang et al. (21) and Liu et al. (24) reported that melatonin improved echocardiographic measurements. Furthermore, studies by Benova et al. (22), Liu et al. (24), Simko et al. (25), Simko et al. (26), Liu et al. (33), Repova et al. (35), and Chen et al. (37) showed that melatonin had a positive effect on hemodynamic variables. In addition, we observed that the effects of melatonin were not significantly different from those reported in the study by Chaudagar et al. (28). These findings indicated the substantial benefit of using melatonin to stabilize hemodynamic parameters. Moreover, Zhu et al. (32) and Chen et al. (37) revealed that melatonin improved left ventricular cardiac function (Figure 2, Table 5).

Zhang et al. (21), Liu et al. (24), Simko et al. (26), Salmanoglu et al. (29), Zhu et al. (32), and Chen et al. (37) revealed that melatonin had positive effects on the rate of apoptosis (Table 5). Melatonin showed positive effects in studies examining western blotting of various proteins and quantitative reverse transcription-polymerase chain reaction (qRT-PCR), including those by Zhang et al. (21), Benova et al. (22), Chen et al. (37), Liu et al. (31), and Liu et al. (24) (Table 5). Drobnik et al. (34) and Repova et al. reported that melatonin reportedly reduced collagen deposition (35) (Table 5). Immunohistochemical analyses were performed by Zhang et al. (21), Stacchiiotti et al. (27), Cheng et al. (30), Liu et al. (33), Drobnik et al. (34), and Cheng et al. (37), revealing that melatonin consistently yielded a positive result (Table 5). Melatonin showed benefits in biometric analyses, as determined by Benova et al. (22), Simko et al. (25), and Chaudagar et al. (28) (Table 5).

Furthermore, melatonin showed beneficial effects on autophagosome evaluation, lactose dehydrogenase measurements, angiotensin, and aldosterone, nitric oxide levels, and mitochondrial analysis, as determined by Zhang et al. (21), Chen et al. (23), Simko et al. (25), Liu et al. (31), Liu et al. (33), Chen et al. (37), and Petrosillo et al. (38) (Table 5).

The meta-analysis revealed a statistically significant decrease in infarct size (MD -20.37 [-23.56, -17.18]). However, there was no statistical difference in systolic pressure between articles analyzed (MD -1.75 [-5.47, 1.97]). In articles analyzing lactate dehydrogenase, a statistically significant decrease in the levels of this enzyme was noted in animals in melatonin groups (MD -4.61 [-6.83, -2.40]). With regard to the ejection fraction, two articles showed improvement in melatonin-treated groups. Another study analyzed the influence of melatonin in infarcted animals with the same ejection fraction; however, this parameter was not statistically significant in the meta-analysis (MD -8.12 [-9.56, -6.69]) (Figure 3).

**Figure 4:**
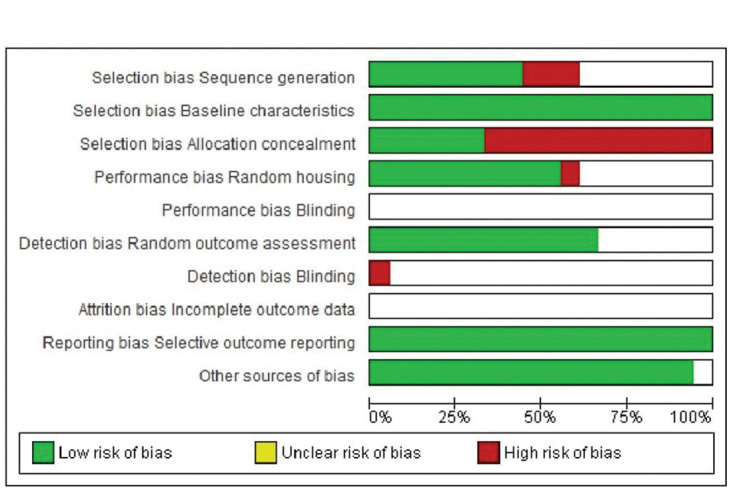
Risk of bias graph: review of authors’ judgment regarding each risk of bias item presented as percentages across all included studies.

**Figure 5:**
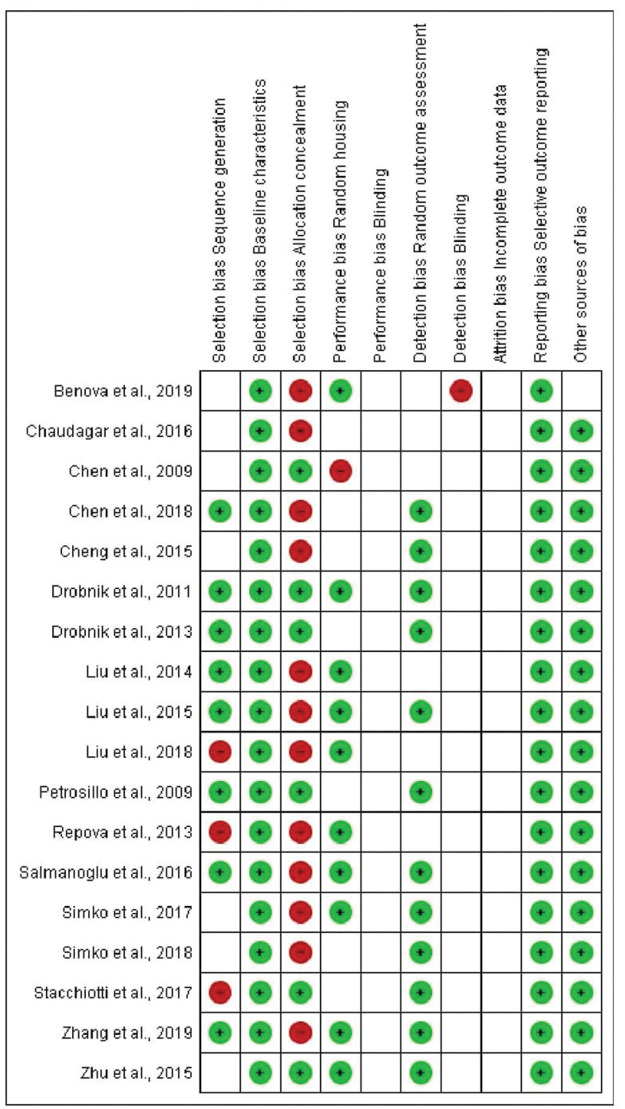
Risk of bias summary: review of authors’ judgment regarding each risk of bias item for each included study.

In terms of selection bias, the results were well-balanced between low risk, no clear risk, and high risk of bias. All studies presented a low risk of bias in the baseline variable characteristics. On analyzing allocation concealment, most selected articles had a high risk, and a little less than half presented a low risk of bias. The randomization parameter was also fairly balanced between low risk, no clear risk, and high risk of bias. On analyzing random outcome assessment, most studies (more than 50%) had a low risk of bias, and some presented an unclear risk of bias. On analyzing blinding bias, most articles were unclear as to whether investigators were blinded. The articles presented a low risk of bias in the results of incomplete outcome data (Figure 4 and 5).

## DISCUSSION

This systematic review revealed that melatonin has various beneficial effects on the cardiovascular system; these effects include decreased infarct size, improved cardiac function and cellular oxidation functions, reduced apoptosis, and healthier cellular histomorphology.

In the present review, studies that analyzed echocardiographic measures exhibited melatonin benefits such as decreased infarct size, improved ejection fractions, improved systolic and diastolic diameters, and ameliorated recovery rates of cardiac function (24), while also favoring the treatment of cardiac hypertrophy and hearts that experienced myocardial infarction, ischemia, or reperfusion (21-38). On analyzing hemodynamic and biometric variables, melatonin appeared to confer significant benefits, such as improvements in systolic pressure, positive pressure derivative, lower left ventricular end-diastolic pressure, reduced left ventricular weight in relation to the total heart weight, and improved lung water content (22-25,28,35,38). Experimental models of obesity, hypertension, and other cardiovascular diseases reinforce the scientific practice of adopting animal models and assessing results prior to human application. These preclinical results indicate the effect of melatonin on the examined cardiovascular diseases.

Reportedly, melatonin is an important anti-apoptotic agent in various tissues, reducing calcium uptake, mitigating reactive oxygen species generation, and decreasing the levels of pro-apoptotic proteins, such as Bax (39). In addition, melatonin destabilizes hypoxia-induced hypoxia-inducible factor (HIF)-1α protein expression. Moreover, melatonin suppresses HIF-1α transcriptional activity under hypoxic conditions, resulting in vascular endothelial growth factor expression (40). Melatonin also confers anti-inflammatory effects on the cardiovascular system (41). Furthermore, a systematic review and recent meta-analysis have identified that melatonin supplementation facilitates blood pressure regulation (42).

Melatonin has substantial benefits in the heart, involving various proteins (including superoxide dismutase [SOD], catalase [CAT], and glutathione peroxidase [Gpx]), while also improving the apoptosis rate. These findings were determined using several techniques, including western blotting analysis of BCL and Bx expression and the TUNEL assay, which measured the decrease in the level of apoptosis in myocardial cells when melatonin was added (23-25,29,32). Other important variables analyzed following melatonin administration in the cardiovascular system were lactate dehydrogenase levels, mitochondrial analysis, lipid peroxidation, glycosaminoglycan, collagen level reduction, culture measurements, the antioxidant action of cells, opening gradient of mitochondrial channels, improvement in vasoconstriction, low-density lipoprotein (LDL), high-density lipoprotein (HDL), and cholesterol level measurements, nitric oxide synthase level measurements, histomorphometric evaluations, determination of hydroxyproline levels, and assessment of autophagosomes (21-38).

The main novelty of this study is that it highlights the benefits assimilated by melatonin in experimental models of myocardial infarction, such as improved ejection fraction. Apart from limitations such as differences between animal organisms and humans, experimental research in Brazil is often restricted due to limited funding for animal studies when compared with human trials. In addition, results from animal studies fail to precisely correlate with the experience of testing melatonin or other substances in an environment that differs from the human body. Another limitation that must be considered is the nature of systematic reviews, which examine non-published data and data previously published by other authors, thus hindering novel scientific findings.

## CONCLUSION

Notably, this systematic review is based on animal experiments. Melatonin may impact the cardiovascular system, including experimental myocardial infarction, and further studies are necessary to determine its use in clinical settings for treating cardiovascular diseases.

## AUTHOR CONTRIBUTIONS

Veiga ECA contributed substantially to the study conception and design, definition of intellectual content, was involved in literature search, data analysis, statistical analysis, and manuscript preparation, drafting and critical review for important intellectual content, and approved the final manuscript version to be published. Simões RS, Caviola LL, Abreu LC and Cavalli RC were involved in data analysis and statistical analysis, manuscript drafting and critical review for important intellectual content, and approved the final manuscript version to be published. Cipolla-Neto J, Baracat EC and Soares Junior JM substantially contributed to the study conception and design, definition of intellectual content, were involved in manuscript preparation, drafting and critical review for important intellectual content, and approved the final manuscript version to be published.

## Figures and Tables

**Figure 3a:**
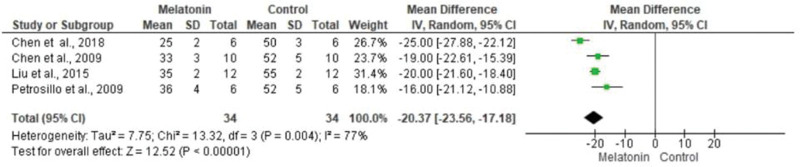
Metanalysis of infarct size measurement by echocardiography (% left ventricular).

**Figure 3b:**
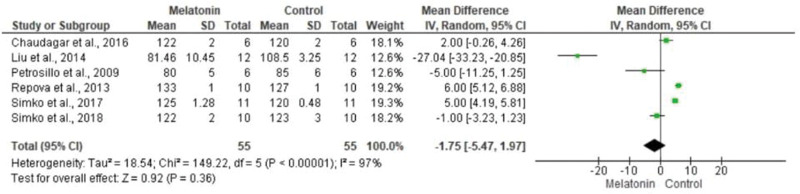
Metanalysis of systolic blood pressure (mmHg).

**Figure 3c:**
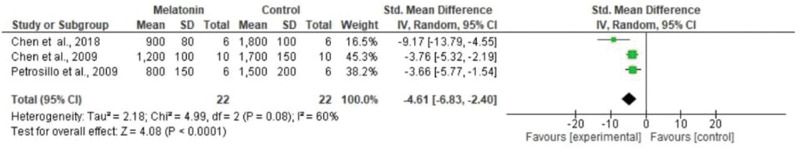
Metanalysis of lactate dehydrogenase (U/L).

**Figure 3d:**
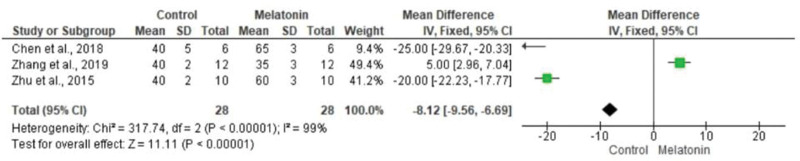
Metanalysis of ejection fraction measured by echocardiography (% left ventricular).

**Table 1:** Study characteristics of selected control experimental studies assessing melatonin and the cardiovascular system.

Authors	Animal type	Animal race	Age (months)	Weight	Induction model	Site injury
Zhang et al. (21)	Mice	C57/B6	-	-	Sepsis-induced cardiac dysfunctional	Cardiovascular system
Benova et al. (22)	Rat	Wistar	9 months	-	Obesity	Cardiovascular system
Chen et al. (23)	Rat	Sprague-Dawley	-	200-250 g	Myocardial ischemia reperfusion	Myocardial tissue
Liu et al. (24)	Mice	C57/B6	6 months	-	Myocardial infarction	Heart
Simko et al. (25)	Rat	Wistar	3 months	-	hypertension	Cardiovascular system
Simko et al. (26)	Rat	Wistar	3 months		hypertension	Cardiovascular system
Stacchiotti et al. (27)	Mice	B6.V^LEAN^/OlaHsd and B6.V-Lep^ob^/OlaHsd	4 weeks	-	Obesity	Mitochondria of cardiomyocyte
Chaudagar et al. (28)	Rat	Wistar	8 months	-	hypertension	Cardiovascular system
Salmanoglu et al. (29)	Rat	Wistar	-	250-350 g	Diabetic	Liver tissue
Cheng et al. (30)	Rabbits	New Zealand	4 weeks	2.0-2.5 kg	Atherosclerosis	Aorta
Liu et al. (31)	Rat	Sprague-Dawley	3 months	280-360 g	Myocardial ischemia reperfusion	Myocardial tissue
Zhu et al. (32)	Rat	Sprague-Dawley	10 weeks	250 g	Myocardial infarction	Heart
Liu et al. (33)	Rat	-	-	350-400 g	Myocardial ischemia reperfusion	Heart
Drobnik et al. (34)	Rat	Wistar	4 weeks	290-320 g	Myocardial infarction	Heart
Repova et al. (35)	Rat	Wistar	3 months		hypertension	Cardiovascular system
Drobnik et al. (36)	Rat	Wistar	-	300-330 g	Myocardial infarction	Heart
Chen et al. (37)	Mice	Mice Gpx^-/-^ C57BL/6	-	-	Myocardial ischemia reperfusion *in vitro*	Heart
Petrosillo et al. (38)	Rat	Wistar	-	250-330 g	Myocardial ischemia reperfusion	Heart

**Table 2:** Characteristics (samples size, number of groups, number of animals/groups, dependent variables) of selected experimental studies assessing the effects of melatonin and the cardiovascular system.

Authors	Sample size	Number of groups	Number of animals/groups	Melatonin administration	Melatonin doses	Dependent variables
Zhang et al. (21)	24	4	6	Intraperitoneal injection	30 mg/kg	Echo, histological analysis, creatinine kinase measurement, TUNEL analysis, western blotting.
Benova et al. (22)	48	4	12	Drinking water	10 mg of melatonin was dissolved in 100 mL of water for 8 weeks	Heart function in Langendorff perfusion, western blot, real-time PCR,
Chen et al. (23)	30	5		Intraperitoneal at the reperfusion	20 mg/kg	Echo, IS2, lactate dehydrogenase release, CMEC measurement *in vitro* IRI assay, western blotting, qRT-PCR, and detection of autophagosomes.
Liu et al. (24)	18	3	6	Gavage	50 mg/kg	Echo, histological analysis, PCR, western blot, CTRP3 detection.
Simko et al. (25)	40	4	10	Water consumption was 12-13 mL/100 g of body weight	10 mg of melatonin was dissolved in 100 mL of water for 4 weeks	Hemodynamics measures, biometric analysis, determination of hydroxyproline, angiotensin, and aldosterone analysis.
Simko et al. (26)	66	6	11	Drinking water adjustment to daily water consumption to ensure the correct dosage	10 mg/kg/ day for 6 weeks	Hemodynamics measures, determination of hydroxyproline, NO synthase activity, oxidative load measurement, and western blotting of NF-_Κ_B.
Stacchiotti et al. (27)	40	4	10	5^th^ to 13^th^ weeks of life/drinking water	100 mg/kg/day for 8 weeks	Histomorphometric evaluations, nuclear cardiomyocyte morphometry, mitochondrial and immunohistochemical analysis.
Chaudagar et al. (28)	24	4	6	Drinking water	10 mg/kg/day for 67 days	Hemodynamics measures, biometric analysis, and NO assays.
Salmanoglu et al. (29)	35	6	-	Oral gavage	10 mg/kg/day for 2 weeks	Vasocontractile response, measurement of total cholesterol, LDL, HDL, glucose, NO, and insulin, MDA assay, and tissue antioxidant levels.
Cheng et al. (30)	60	3	20	-	20 mg/kg for 4 weeks	Immunohistochemical analysis, HE staining, western blot analysis, and qRT-PCR.
Liu et al. (31)	60	5	12	Intravenous injection immediately after reperfusion	10 mg/kg	IF2, myocardial ultrastructure, western blotting and determination of the opening degree of MPTPs.
Zhu et al. (32)	-	-	-	Melatonin stem cells were treated for 24 hours	5 µM	Measurements of cell culture antioxidant properties, apoptosis, analysis of paracrine factors, LV functions, histology.
Liu et al. (33)	60	6	12	Intraperitoneal injection	Group I: 2.5 mg/kg, Group II: 5 mg/kg, Group III: 10 mg/kg	Hemodynamics measures, apoptosis, electron microscope examination, analysis on mitochondria.
Drobnik et al. (34)	21	3	7	Drinking water for 6 weeks	10 mg/kg	Collagen determination, estimation of glycosaminoglycans, electron microscope examination.
Repova et al. (35)	40	4	10	Drinking water for 6 weeks	10 mg/kg	Collagen determination, hemodynamics measures.
Drobnik et al. (36)	60	5	12	Intraperitoneal injection for 4 weeks	Group 1: 300 µg/100 g b.w. Group 4: 3 mg/100 g.b.w. Group 5: 1.5 mg/100 g.b.w.	Estimation of lipid peroxidation, collagen determination, estimation of glycosaminoglycans.
Chen et al. (37)	-	-	-	Intraperitoneal injection 30 min before harvesting the hear for *in vitro* preparation	150 µg/kg	Cardiac function, hemodynamics measures, lactate dehydrogenase released, apoptosis, immunohistochemistry.
Petrosillo et al. (38)	42	6	7	Krebs-Henseleit solution for isolated heart	50 µM	Infarct size, lactate dehydrogenase released, hemodynamics measures, analysis on mitochondria.

IS1, measurement of infarct size by echocardiography; IS2, measurement of infarct size by Evans Blue or tetrazolium; echo, echocardiography measurements; CMEC, cardiac microvascular endothelial cells; IRI, ice recrystallization inhibition; CTRP3, C1q TNF Related Protein 3; NO, nitric oxide; LDL, low-density lipoprotein; HDL, high-density lipoprotein; MDA, malondialdehyde; qRT-PCR, quantitative reverse transcription-polymerase chain reaction; LV, left ventricular; MPTP, mitochondrial permeability transition pore; NF-_Κ_B, Nuclear factor-kappa B; g.b.w., gross body weight; HE, hematoxylin-eosin.

**Table 3:** Most frequent recommendations appearing in preclinical research guidelines for *in vivo* animal experiments [Hendersen et al. (18)].

Validity type	Recommendation Category	Examples
Internal	Choice of sample size	Power calculation, larger samples sizes
	Randomized allocation of animals to treatment	Various methods of randomization
	Blinding of outcome assessment	Blinded measurement or analysis
	Flow of animals through an experiment	Recording animals excluded from treatment through to analysis
	Selection of appropriate control groups	Using negative, positive, concurrent, or vehicle control groups
	Study of dose-response relationships	Testing above and below optimal therapeutic dose
Construct	Characterization of animal properties at baseline	Characterizing inclusion/exclusion criteria, disease severity, age or sex
	Matching model to the human manifestation of the disease	Matching mechanism, chronicity or symptoms
	Treatment response along a mechanistic pathway	Characterizing pathway in terms of molecular biology, histology, physiology or behavior
	Matching outcome measures to clinical settings	Using functional or non-surrogate outcome measures
	Matching model to the age of patients in clinical settings	Using aged or juvenile animals
External	Replication in different models of the same disease	Different transgenic strains or lesion techniques
	Independent replication	Different investigators or research groups
	Replication in different species	Rodents and nonhuman primates
Research program	Inter-study standardization of experimental design	Coordination between independent research groups

**Table 4:** Most frequent recommendations in preclinical research guidelines for *in vivo* animal experiments [Henderson et al. (18)].

Validity type	Recommendation Category	Studies	n (Percent of guidelines Citing)
Internal	Choice of sample size	Zhang et al. (21); Benova et al. (22); Simko et al. (25); Simko et al. (26); Stacchiotti et al. (27); Cheng et al. (30); Liu et al. (31); Liu et al. (33); Repova et al. (35); Drobnik et al. (36); Petrosillo et al. (38).	61.11%
	Randomized allocation of animals to treatment	Zhang et al. (21); Chen et al. (23); Simko et al. (25); Simko et al. (26); Salmanoglu et al. (29); Cheng et al. (30); Liu et al. (31); Liu et al. (33); Drobnik et al. (34); Repova et al. (35); Drobnik et al. (36).	61.11%
	Blinding of outcome assessment	Zhang et al. (21); Benova et al. (22); Chen et al. (23); Simko et al. (25); Liu et al. (24); Simko et al. (26); Stacchiotti et al. (27); Chaudagar et al. (28); Salmanoglu et al (29); Cheng et al. (30); Liu et al. (31); Zhu et al. (32); Liu et al. (33); Drobnik et al. (34); Repova et al. (35); Drobnik et al. (36); Chen et al. (37); Petrosillo et al. (38).	100%
	Flow of animals through an experiment	-	-
	Selection of appropriate control groups	Zhang et al. (21); Chen et al. (23); Simko et al. (25); Simko et al. (26); Stacchiotti et al. (27); Chaudagar et al. (28); Salmanoglu et al. (29); Cheng et al. (30); Liu et al. (31); Liu et al. (33); Drobnik et al. (34); Repova et al. (35); Drobnik et al. (36); Petrosillo et al. (38).	77.77%
	Study of dose-response relationships	Chen et al. (23); Simko et al. (25); Simko et al. (26); Chaudagar et al. (28); Cheng et al. (30); Liu et al. (31); Liu et al. (33); Drobnik et al. (34); Repova et al. (35); Drobnik et al. (36); Petrosillo et al. (38).	61.11%
Construct	Characterization of animal properties at baseline	Zhang et al (21); Benova et al. (22); Chen et al. (23); Simko et al. (25); Liu et al (24); Simko et al. (26); Stacchiotti et al. (27); Chaudagar et al. (28); Salmanoglu et al. (29); Cheng et al. (30); Liu et al. (31); Zhu et al. (32); Liu et al. (33); Drobnik et al. (34); Repova et al. (35); Drobnik et al. (36); Chen et al. (37); Petrosillo et al. (38).	100%
	Matching model to the human manifestation of the disease	Zhang et al. (21); Benova et al. (22); Chen et al. (23); Simko et al. (25); Simko et al. (26); Salmanoglu et al. (29); Cheng et al. (30); Liu et al. (31); Zhu et al. (32); Liu et al. (33); Drobnik et al. (34); Repova et al. (35); Drobnik et al. (36); Chen et al. (37); Petrosillo et al. (38).	88.88%
	Treatment response along a mechanistic pathway	Zhang et al. (21); Benova et al. (22); Chen et al. (23); Simko et al. (25); Liu et al. (24); Simko et al. (26); Stacchiotti et al. (27); Chaudagar et al. (28); Salmanoglu et al. (29); Cheng et al. (30); Liu et al (31); Zhu et al. (32); Liu et al. (33); Drobnik et al. (34); Repova et al. (35); Drobnik et al. (36); Chen et al. (37); Petrosillo et al. (38).	100%
	Matching outcome measures to clinical settings	Chen et al. (23); Simko et al. (25); Simko et al. (26); Stacchiotti et al. (27); Chaudagar et al. (28); Salmanoglu et al. (29); Cheng et al. (30); Liu et al. (31); Liu et al. (33); Repova et al. (35); Chen et al. (37); Petrosillo et al. (38).	66.66%
	Matching model to the age of patients in clinical settings	Zhang et al. (21); Benova et al. (22); Chen et al. (23); Simko et al. (25); Liu et al. (24); Simko et al. (26); Stacchiotti et al. (27); Chaudagar et al. (28); Salmanoglu et al. (29); Liu et al. (31); Zhu et al. (32); Liu et al. (33); Drobnik et al. (34); Repova et al. (35); Drobnik et al. (36); Chen et al. (37); Petrosillo et al. (38).	100%
External	Replication in different models of the same disease	-	-
	Independent replication	Zhang et al. (21); Chen et al. (23); Simko et al. (25); Simko et al. (26); Stacchiotti et al. (27); Chaudagar et al. (28); Salmanoglu et al. (29); Cheng et al. (30); Zhu et al. (32); Drobnik et al. (34); Repova et al. (35); Drobnik et al. (36); Chen et al. (37); Petrosillo et al. (38).	77.77%
	Replication in different species	Zhang et al. (21); Benova et al. (22); Chen et al. (23); Simko et al. (25); Simko et al. (26); Stacchiotti et al. (27); Chaudagar et al. (28); Salmanoglu et al. (29); Liu et al. (31); Zhu et al. (32); Liu et al. (33); Drobnik et al. (34); Repova et al. (35); Drobnik et al. (36); Chen et al. (37); Petrosillo et al. (38).	88.88%
Research program	Inter-study standardization of experimental design	Simko et al. (25); Simko et al. (26); Stacchiotti et al. (27); Chaudagar et al. (28); Zhu et al. (32); Repova et al. (35); Chen et al. (37).	38.88%

**Table 5:** Study characteristics of selected controlled animal studies assessing melatonin and the cardiovascular system.

Authors	Assessments					
Zhang et al. (21)	S	S	S	S	S	S
	Echocardiography measurements	Apoptosis analysis	Western blotting	Creatinine kinase measurement	Immunohistochemical analysis	Detection of autophagosomes
Benova et al. (22)	S	S	S	S	NS	
	Biometric analysis	Western blotting	qRT-PCR	Hemodynamics measures		
Chen et al. (23)	S	S	S	S	S	S
	Measurements of the infarct size	Measurement of lactate dehydrogenase	Measures of CMEC *in vitro* IRI assay	Western blotting	qRT-PCR	Detection of autophagosomes
Liu et al. (24)	S	S	S	S	S	
	Echocardiography measurements	Hemodynamics measurements	Apoptosis analysis	Western blotting	qRT-PCR	
Simko et al. (25)	S	S	NS	S	NS	
	Biometric analysis	Hemodynamics measures	Determination of hydroxyproline	Angiotensin analysis	Aldosterone analysis	
Simko et al. (26)	NS	S	S	S	S	
	Hemodynamics measures	Determination of hydroxyproline	NO synthase activity	Oxidative load	Measurement and western blotting of NF-_Κ_B	
Stacchiotti et al. (27)	S	S	S	S		
	Histomorphometrically evaluations	Nuclear cardiomyocyte morphometric	Mitochondrial analysis	Immunohistochemical analysis		
Chaudagar et al. (28)	NS	S	S			
	Hemodynamics measures	Biometric analysis	NO assays			
Salmanoglu et al. (29)	NS	NS	S	S	NS	
	Vasocontractile response	Measures of total cholesterol, LDL, HDL	NO assays	MDA assay	Measurements of tissue antioxidant levels	
Cheng et al. (30)	S	S	S			
	Immunohistochemical analysis	Western blotting	qRT-PCR			
Liu et al. (31)	S	S	S			
	Measurements of the infarcted size	Western blotting	Determination of the opening degree of MPTPs			
Zhu et al. (32)	S	S	S			
	Measurements of cell cultures antioxidant properties	Apoptosis analysis	LV functions			
Liu et al. (33)	S	S	S	S		
	Hemodynamics measures	Apoptosis analysis	Electron microscope examination	Analysis on mitochondria		
Drobnik et al. (34)	S	S	S			
	Determination of collagens	Determination of glycosaminoglycans	Electron microscope examination			
Repova et al. (35)	S	S				
	Hemodynamics measures	Determination of collagen				
Drobnik et al. (36)	S	NS	S			
	Estimation of lipid peroxidation	Determination of collagen	Determination of glycosaminoglycans			
Chen et al. (37)	S	S	S	S	S	
	Cardiac function	Hemodynamics measures	Lactate dehydrogenase	Apoptosis analysis	Immunohistochemistry	
Petrosillo et al. (38)	S	S	S	S		
	Measurements of the infarct size	Lactate dehydrogenase	Hemodynamics measures	Analysis on mitochondria		

S, statistically significant; NS, not significant; CMEC, cardiac microvascular endothelial cells; IRI, ice recrystallization inhibition; qRT-PCR, quantitative reverse transcription-polymerase chain reaction; NO, nitric oxide; NF-_Κ_B, Nuclear factor-kappa B; LDL, low-density lipoprotein; HDL, high-density lipoprotein; MDA, malondialdehyde; LV, left ventricular.
